# Research progress of comprehensive therapy dominated by “massage” in the treatment of cervical spondylotic radiculopathy: A review

**DOI:** 10.1097/MD.0000000000046519

**Published:** 2025-12-12

**Authors:** Ruiqian Guan, Yongpeng Mi, Yanhong Ban, Haichun Zhou

**Affiliations:** a Heilongjiang University of Chinese Medicine, Harbin, Heilongjiang, China; b Second Affiliated Hospital of Heilongjiang University of Chinese Medicine, Harbin, Heilongjiang, China; c Shijiazhuang Hospital of Traditional Chinese Medicine, Shijiazhuang, Hebei Province, China; d Fourth Affiliated Hospital of Heilongjiang University of Chinese Medicine, Harbin, Heilongjiang, China.

**Keywords:** acupuncture, massage, nerve root type cervical spondylosis, review

## Abstract

Cervical spondylotic radiculopathy is the most common type of cervical spondylosis. It is a disease caused by cervical nerve root compression due to various reasons. Clinically, neck muscle stiffness, pain and other activity disorders, or accompanied by dizziness, headache and other symptoms, bring great inconvenience to patients. Clinical studies have confirmed that the use of massage-based treatment, clinical achieved satisfactory clinical results. This article will take ‘ massage ‘ as the leading combination of acupuncture, traditional Chinese medicine fumigation, traction, oral administration of traditional Chinese medicine, fire therapy, acupotomy, exercise therapy and other therapies to treat cervical spondylosis of nerve root type, so as to guide clinical practice.

## 1. Introduction

**Figure 1.**
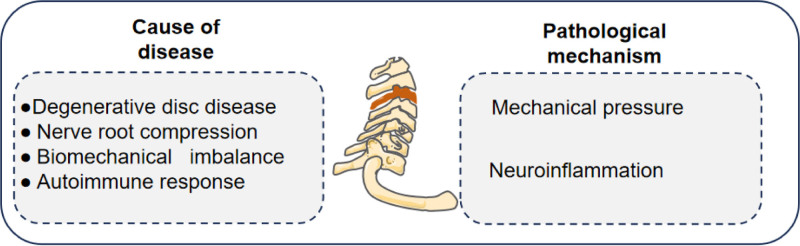
Pathogenesis of nerve root type cervical spondylosis.

Cervical spondylosis is due to the degeneration of the cervical intervertebral disc or the instability of the cervical spine due to the imbalance of the dynamic and static forces of the cervical spine. It is manifested as a series of clinical symptoms such as neck and shoulder muscle stiffness, pain, dizziness, headache, or radioactive pain and numbness of one or both upper limbs.^[1–3]^ Among them, cervical spondylotic radiculopathy accounts for more than half, accounting for the most^.[4,5]^ The clinical manifestations of cervical spondylotic radiculopathy are mainly neck and shoulder pain, radiation pain and numbness of upper limbs or fingers caused by nerve root compression.^[6,7]^ A large number of clinical data show that the ‘ massage ‘ as the leading, the use of 2 or more than 2 kinds of comprehensive therapy in the treatment of cervical spondylosis of nerve root type curative effect is remarkable.^[8]^ The literature review in this area is as follows (refer to Figure 1).

## 2. Tuina therapy

Xie Zhaohua^[9]^ used traditional Chinese medicine massage to treat patients with cervical spondylosis of nerve root type. The treatment methods include manual release, traction, reduction, massage and so on. It was found that TCM massage can improve nerve root compression and promote blood circulation by correcting the dislocation of cervical facet joints, and achieve analgesic effect by relieving muscle spasm, which can significantly improve the clinical symptoms of patients with cervical spondylotic radiculopathy. Guo Zhibin et al^[10]^ performed massage therapy on patients with cervical spondylotic radiculopathy and found that the balance of muscles and bones can promote local blood circulation, remove metabolites, accelerate the absorption of local inflammatory substances, and improve the local internal environment.^[11,12]^ Tuina has the functions of promoting qi and activating blood, relaxing tendons and dredging collaterals, relieving spasm and relieving pain. Tuina manipulation is guided by the theory of meridians and collaterals, and manipulation is performed on the neck, shoulder and upper limbs. Tuina conforms to the theory of holistic view of traditional Chinese medicine, regulates the whole body meridian qi and qi and blood, and restores the physiological function of viscera. Therefore, the massage treatment of cervical spondylosis of nerve root type has a lasting and stable effect, which can fundamentally reduce pain, and is easy to be accepted by patients, comfortable and safe.

## 3. Massage combined with acupuncture therapy

Li Fuqiu^[13]^ used massage combined with acupuncture to treat patients with cervical spondylotic radiculopathy. It was found that massage could relieve the adhesion between cervical nerve root and surrounding soft tissue and relax the neck and surrounding soft tissue. Acupuncture directly stimulates the spinal nerve to achieve analgesic effect, which can activate meridians, activate blood circulation, relieve pain, warm yang and remove impediment. The combined use of the 2, the effect is significantly better than the single therapy.^[14]^ Chen Penghuan^[15]^ found that massage combined with acupuncture and moxibustion can release the tense muscles, expand the intervertebral space, and promote the mechanical balance of the cervical spine. Combined with acupuncture to stimulate specific acupoints in the neck, to achieve the effect of dispelling wind, dispelling cold and relieving pain, promoting blood circulation and removing blood stasis and swelling. The combination of the 2 has a significant therapeutic effect, and the course of treatment is shortened accordingly. Wang Hao^[16]^ used massage combined with electroacupuncture treatment, and found that massage can improve muscle tension by relaxing the muscles of the neck and shoulder, and absorb and dissipate edema by promoting blood flow velocity; combined with electroacupuncture treatment, it can dispel wind, disperse cold and dredge collaterals, and its therapeutic effect is remarkable. Traditional Chinese medicine has used acupuncture and moxibustion to treat cervical spondylosis for a long time, and the curative effect is positive. Acupuncture and moxibustion can obviously relieve the adhesion of neck and shoulder muscles, promote the smooth flow of qi and blood in meridians, and can also drive out evil spirits to achieve the purpose of no pain in general. Tuina combined with acupuncture and moxibustion in the treatment of cervical spondylosis of nerve root type is not only better than single treatment, but also can shorten the course of treatment, which is worthy of popularization and application.

## 4. Tuina combined with traditional Chinese medicine fumigation therapy

Tian Jitao et al^[17]^ used massage and bonesetting combined with traditional Chinese medicine fumigation treatment, and found that traditional Chinese medicine fumigation directly acts on the treatment site through heat convection and penetration, promotes the utilization of drugs, and directly passes through the acupoints. Combined with bone-setting manipulations such as relaxing tendons and dredging collaterals, promoting blood circulation and removing blood stasis, detumescence and pain relief, and correcting dislocation, it can quickly and effectively relieve the symptoms of patients with cervical spondylotic radiculopathy, and the curative effect is remarkable. Cui et al^[18]^ gave traditional Chinese medicine fumigation combined with massage therapy to patients with cervical spondylotic radiculopathy, and found that traditional Chinese medicine fumigation therapy promoted telangiectasia, and the drug penetrated the skin through dilated pores, which not only accelerated blood circulation, but also accelerated the excretion of local pathological products. In addition, massage spasmolysis and pain relief, correction of dislocation, the combination of the 2 in the treatment of nerve root type cervical spondylosis clinical efficacy is satisfactory. Traditional Chinese medicine fumigation mostly uses traditional Chinese medicine that promotes blood circulation, warms the meridians and dispels cold. It opens sweat pores through hot steam, penetrates traditional Chinese medicine into the lesion site, and improves muscle numbness, tension, spasm and pain. Moreover, traditional Chinese medicine fumigation takes into account the difficulty of oral administration of traditional Chinese medicine in some patients, expanding the scope of application of patients. However, the simple use of traditional Chinese medicine fumigation is effective, and the improvement of patients ‘ pain is not ideal. Therefore, it is necessary to actively combine massage, acupuncture and other therapies to make the treatment effect more thorough and more secure.

## 5. Tuina combined with traction therapy

Liu Xinghe^[19]^ used massage, acupuncture combined with traction therapy, and found that traction changed the curvature of the cervical spine by expanding the intervertebral space, thereby accelerating the blood flow velocity of the cervical spine and reducing pain. Combined with acupuncture and moxibustion for dispelling wind and cold, activating blood circulation and dredging collaterals, massage for relieving muscle stiffness and spasm can effectively improve the clinical symptoms of patients with cervical spondylosis of nerve root type. Jiang^[20]^ used massage, acupuncture combined with cervical traction treatment, found that massage can correct cervical facet joint disorder, release neck and shoulder muscle stiffness and adhesion; acupuncture and moxibustion can promote blood circulation, relieve pain and dredge collaterals, promote blood circulation and inflammation absorption and dissipation in neck and shoulder; combined traction can relieve the stimulation of the nerve root by regulating the imbalance of the static and dynamic forces of the cervical spine, and improve the stability of the cervical spine by improving the activity of the cervical spine. Through the combined treatment, the clinical symptoms of patients with cervical spondylotic radiculopathy can be significantly improved. If the clinical efficacy of traction alone in the treatment of cervical spondylosis of nerve root type is not significant, it is necessary to use traction combined with massage and acupuncture to treat cervical spondylosis of nerve root type according to the actual situation and the actual condition of the patient. The curative effect is significant and achieves synergistic effect.

## 6. Tuina combined with traditional Chinese medicine oral therapy

Wang Dazhi et al^[21]^ used acupuncture and massage combined with traditional Chinese medicine for treatment. The traditional Chinese medicine selected Erchen Decoction to add and subtract. The effect was to promote qi and phlegm, promote blood circulation, relax muscles, loosen adhesions, and promote blood circulation. Xie Min^[22]^ selected massage, acupuncture combined with traditional Chinese medicine oral Gegen Decoction treatment, the efficacy of Gegen Decoction for promoting blood circulation and removing blood stasis, qi and pain; combined with massage, it can relax muscles, improve blood circulation, relax muscles and activate blood; acupuncture can stimulate acupoints, warm meridians, dispel cold and remove arthralgia. The combined application can effectively relieve pain and improve the quality of life of patients.

## 7. Tuina combined with fire therapy

Cheng Jintao^[23]^ selected acupuncture and massage combined with fire therapy. Fire therapy is a large area of moxibustion therapy. It promotes local blood circulation through alcohol combustion, which can relieve the symptoms of neck and shoulder pain and upper limb numbness in patients with cervical spondylotic radiculopathy. It is beneficial to relieve muscle tension and relieve pain in patients. Combined with acupuncture and massage therapy, it can clearly improve the clinical symptoms of patients. However, fire therapy has many contraindications and precautions that need to be applied in accordance with the will. Some patients are afraid of flame, and there are many limitations in the application, which is not suitable for large-scale application.

## 8. Tuina combined with needle knife therapy

Lin Wenchuan^[24]^ selected acupotomy combined with massage therapy, and found that acupotomy treatment can release, peel scars or contractures at the lesion site, reduce the traction of the diseased vertebral body, reduce nerve root edema, improve local microcirculation, increase blood flow, and relieve external mechanical factors. Combined with massage to dredge the internal meridians, restore joint dislocation. The combination of the 2 can improve the tension of the trapezius muscle, restore the biomechanical balance of the cervical spine, and fundamentally treat cervical spondylosis. Zhang Hu^[25]^ et al used acupotomy to treat patients with cervical spondylosis of nerve root type, and found that the stimulation of acupotomy to the surrounding tissue of the lesion was significantly greater than that of filiform needle acupuncture, eliminating the disease knot, improving the muscle tension of the trapezius muscle, restoring the mechanical balance, and fundamentally treating cervical spondylosis. Wang Jianguang^[26]^ treated with acupotomy under the guidance of ultrasound, which is different from the blind treatment of acupotomy only based on the experience and feeling of doctors. With the intuition and accuracy of modern scientific and technological ultrasound images, it presents real-time dynamic images and is more easily accepted by patients. However, the sterility of ultrasonic probes, the application of higher-level equipment, medical expenses and costs, and standardized operation guidelines have become obstacles to its popularization and application.

## 9. Massage combined with exercises

**Figure 2.**
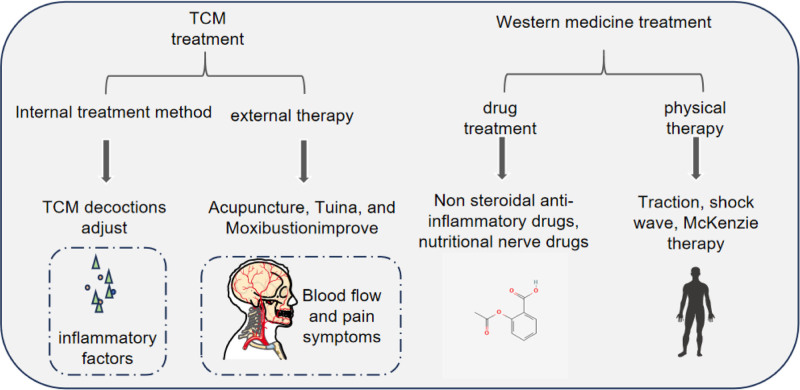
Integrative medicine treatment.

Tu Jinkang^[27]^ and other studies have found that patients with cervical spondylotic radiculopathy adhere to exercise Baduanjin, which can relieve neck and shoulder pain, strengthen the core strength of the neck, and improve the anxiety and depression of patients. The 8-section brocade movement is simple, there is no venue restriction, it can be carried out anytime and anywhere, and there is no dependence on doctors. After learning the movements and precautions, you can practice independently. However, the practice of Baduanjin needs to be adhered to for a long time. It cannot be abandoned halfway. It takes 3 days to fish and 2 days to dry the net (refer to Fig. 2).

## 10. Conclusion

Cervical spondylotic radiculopathy refers to a series of symptoms such as pain, numbness, and dyskinesia due to cervical nerve root compression and cervical stenosis, resulting in cervical curvature, reduced disc stability, bone hyperplasia or edema.^[28,29]^ The main methods of clinical treatment of this disease are surgical treatment and non-surgical treatment. Among them, surgical treatment can directly reduce the mechanical compression of the nerve root and relieve the clinical symptoms, but the surgical trauma is large, the risk is high, the patient’s physical quality is required, and the postoperative bed rest and recovery are required. The time line is long, affecting the patient’s work and life, and it is easy to relapse after surgery, and the cost performance is not high. At present, conservative treatment is still the main clinical treatment. Traditional Chinese medicine massage is one of the most commonly used conservative treatment methods. Traditional Chinese medicine massage treatment of nerve root type cervical spondylosis has obvious clinical effect, and has the advantages of short course of treatment, no adverse reaction, not easy to relapse. When treated by traditional Chinese medicine massage, the main purpose is to relieve spasm and pain, correct dislocation, so as to promote blood circulation and remove blood stasis, improve or relieve the compression of nerves and blood vessels by adjacent tissues. It can effectively relieve muscle pain and promote the circulation of qi and blood in the neck and the whole body. At the same time, the symptoms of cervical spondylotic radiculopathy were improved from 2 aspects of blood flow and muscle tissue. Simple massage therapy only focuses on the release and dredging of muscle soft tissue. The clinical treatment has a long course of treatment and the symptoms are easy to repeat. Acupuncture is based on the theory of zang-fu organs and meridians of traditional Chinese medicine. For the wind, cold, dampness and stasis of cervical spondylosis, it has the effect of dispelling wind, dispelling cold, eliminating dampness and removing blood stasis.^[30]^ The use of acupuncture in the treatment of patients with cervical spondylotic radiculopathy can significantly improve the clinical symptoms of patients, promote blood circulation, and effectively relieve symptoms such as neck and shoulder numbness and radioactive pain^.[31,32]^ Electroacupuncture combined with the dual effects of pulsed electrical stimulation and acupuncture promotes the release of dynorphins from the spinal cord and produces analgesic effects.^[33]^ Traction can relieve neck and shoulder back muscle tension and spasm by expanding the intervertebral space, and restore the dynamic and static mechanical balance of the cervical spine, and can reduce the compression stimulation of the nerve root, accelerate the blood circulation of the neck, and accelerate the absorption and excretion of the pain-causing substances, so as to achieve the effect of relieving pain and swelling.^[34]^ Oral administration of traditional Chinese medicine through syndrome differentiation and treatment, flexible selection of suitable decoction for patients, effectively improve the symptoms of patients with cervical spondylosis.^[35]^ Traditional Chinese medicine fumigation through heat convection and infiltration, direct effect and treatment site, promote the utilization of drugs, through the meridians, is an effective therapy for some patients who do not want to take traditional Chinese medicine. Acupotomy also has the effect of local analgesia and central analgesia^[36]^; it also effectively loosens, peels and cuts the diseased tissue to improve muscle tension and achieve the purpose of ‘ removing the loose ‘. Gongfa such as Baduanjin can significantly reduce neck and shoulder pain and upper limb numbness symptoms, and the long-term effect is significant. In the treatment of patients with cervical spondylotic radiculopathy, we can flexibly match massage, acupuncture, electroacupuncture, traction, traditional Chinese medicine, exercises and other therapies, and select a variety of therapies suitable for the patient ‘s condition, which can significantly improve the patient ‘s clinical symptoms, shorten the course of treatment, and promote the rehabilitation of patients. Due to the development of science and technology, the wide application of mobile phones and computers, bad living habits, or the requirements of long-term desk work, or the influence of geographical environment, cervical spondylosis is prone to repeated attacks and is difficult to heal. The clinical treatment plan lacks standardization and normalization, and lacks observation of long-term efficacy. Therefore, how to give a more perfect treatment plan and a longer-term treatment goal requires the joint efforts of clinicians and researchers.

## Author contributions

**Investigation:** Yongpeng Mi.

**Visualization:** Yanhong Ban.

**Writing – original draft:** Haichun Zhou.

**Writing – review & editing:** Ruiqian Guan.
